# Modeling the calcium spike as a threshold triggered fixed waveform for synchronous inputs in the fluctuation regime

**DOI:** 10.3389/fncom.2015.00091

**Published:** 2015-07-28

**Authors:** Yansong Chua, Abigail Morrison, Moritz Helias

**Affiliations:** ^1^Institute for Advanced Simulation (IAS-6) and Institute of Neuroscience and Medicine (INM-6) and JARA BRAIN Institute I, Jülich Research CentreJülich, Germany; ^2^Faculty of Biology, Albert-Ludwig University of FreiburgFreiburg im Breisgau, Germany; ^3^Bernstein Center Freiburg, Albert-Ludwig University of FreiburgFreiburg im Breisgau, Germany; ^4^Faculty of Psychology, Institute of Cognitive Neuroscience, Ruhr-University BochumBochum, Germany

**Keywords:** calcium spikes, pyramidal neuron, calcium spike induced somatic voltage excursion, calcium spike approximation, calcium spike as threshold triggered fixed waveform

## Abstract

Modeling the layer 5 pyramidal neuron as a system of three connected isopotential compartments, the soma, proximal, and distal compartment, with calcium spike dynamics in the distal compartment following first order kinetics, we are able to reproduce *in-vitro* experimental results which demonstrate the involvement of calcium spikes in action potentials generation. To explore how calcium spikes affect the neuronal output *in-vivo*, we emulate *in-vivo* like conditions by embedding the neuron model in a regime of low background fluctuations with occasional large synchronous inputs. In such a regime, a full calcium spike is only triggered by the synchronous events in a threshold like manner and has a stereotypical waveform. Hence, in such a regime, we are able to replace the calcium dynamics with a simpler threshold triggered current of fixed waveform, which is amenable to analytical treatment. We obtain analytically the mean somatic membrane potential excursion due to a calcium spike being triggered while in the fluctuating regime. Our analytical form that accounts for the covariance between conductances and the membrane potential shows a better agreement with simulation results than a naive first order approximation.

## 1. Introduction

Recent studies have shown that dendritic spikes play a significant role in the functions of neurons *in-vivo* (Sivyer and Williams, 2013; Smith et al., 2013; Grienberger et al., 2014; Palmer et al., 2014). Prior to these *in-vivo* experiments, dendritic spikes, in particular calcium spikes in the layer 5 pyramidal neuron, had already been extensively studied *in-vitro* (Stuart et al., 1997; Larkum et al., 1999a,b, 2001; Williams and Stuart, 2002). Larkum et al. (1999a) showed that when an action potential coincides with a distal current stimulation, the back-propagating action potential couples with the current stimulus to trigger a calcium spike, which then propagates to the soma and triggers further action potentials, resulting in a burst of action potentials. This coupling time window is in the range of 20–30 ms, and the stimulus required to trigger a calcium spike halves when optimally coupled with the first back-propagating action potential. Larkum et al. (2001) subsequently demonstrated that a hyperpolarizing current at the proximal dendrite prevents a calcium spike from triggering action potentials at the soma. Larkum et al. (1999b) also showed that if only action potential bursts alone are to trigger a calcium spike, they have to spike above a certain critical frequency in order to do so. In the above studies, distal synaptic inputs are typically severely attenuated by the time they reach the soma, calling into question their impact on the neuron's firing activities. Larkum et al. (2004) proposed that distal inputs could serve to increase the gain of the pyramidal neuron, accompanied by a change of firing modes from single action potentials to burst firing during coincident somatic and distal inputs. In the accompanying two compartment neuron model, the calcium spike is modeled using first order kinetics to explain this gain modulation.

Building on the above *in-vitro* studies, recent studies investigate the functional significance of the calcium spike. Larkum et al. (2009) used both experiments and simulation studies in NEURON (Carnevale and Hines, 2006) to determine how synchronous local NMDA spikes and synaptic inputs on distal tuft dendrites cooperate to activate calcium spikes which then propagate to the soma. The interaction of these various levels of active dendritic events are reviewed by Major et al. (2013) who conclude that they may enable the pyramidal neuron to function as a multi-layered computational unit, while another review argues that the calcium spike might serve to amplify coincident inputs, so influencing the neuronal output (Spruston, 2008). Larkum (2013) puts forward the hypothesis that calcium spikes serve to bind top-down and bottom-up synaptic inputs, and, in combination with the local micro-circuits, underlie the organizing principle of the cerebral cortex.

It is notable that these hypotheses on the functional role of calcium spikes are based predominantly on observations from *in-vitro* studies. It remains largely unclear whether the observed phenomena would also be observable *in-vivo*. Specifically, can a calcium spike still be triggered by coincident inputs? Assuming it can, under what conditions does it have an impact on the neuron's spiking activity? In the absence of corresponding *in-vivo* experimental protocols, it is a reasonable strategy to apply a theoretical approach supported by numerical simulations to shed light on these questions.

The layer 5 pyramidal neuron has been extensively modeled in NEURON and latest studies try to fit neuron parameters to emulate experimental findings (Hay et al., 2011; Bahl et al., 2012; Almog and Korngreen, 2014). In these studies, the calcium spike is triggered by different kinds of voltage dependent calcium channels such as medium voltage activated (MVA) and high voltage activated (HVA) calcium channels. Almog and Korngreen (2014) also call into question the hypothesis of a calcium hot spot at the distal main bifurcation point proposed by Larkum et al. (2009). They present an alternative hypothesis of a gradient of calcium channels along the apical dendrite to explain calcium spikes and action potential bursts; this has yet to be verified experimentally.

Although these complex neuron models are useful for understanding the biophysical mechanisms of the generation and propagation of calcium spikes, they are less helpful for investigating their functional role, as they are neither sufficiently tractable to allow theoretical analysis nor sufficiently simple to permit efficient simulation in large networks. In this article, we address this problem by developing a neuron model that is amenable to both analysis and efficient simulation whilst still reproducing key *in-vitro* experimental results.

As an initial step, we introduce a three compartment neuron model. The three compartments represent the soma [with basal dendrites], proximal [apical dendrite with oblique proximal dendrites], and distal [distal bifurcation point and tuft dendrites]. The calcium spike is modeled using first order kinetics in the distal compartment (see 2.1). We show that this neuron model is able to reproduce a variety of *in-vitro* experimental results as shown in 3.1 for a single set of parameters.

Although this initial model already represents a considerable simplification over complex biophysical models, it still contains a large number of dynamic variables and is not analytically tractable. We therefore examine the behavior of this parameterized model under different *in-vivo* like conditions to identify the conditions in which a further simplification of a fixed calcium potential waveform triggered by a voltage threshold would be valid. To this end, we embed the neuron model in four different regimes, in which calcium spikes are either triggered by large background fluctuations or large synchronous inputs (**Figure 2**). We determine that the proposed model reduction is a good approximation in the regime of low background fluctuation with synchronous events resulting in large coincident inputs. Both the threshold and waveform are dependent on the time constants of the synchronous inputs which can be empirically obtained from the calcium spike modeled using first order kinetics (**Figure 3**). We show that despite the reduction in free parameters, the model can reproduce the same experimental results as the more complex model (**Figure 8**).

As the reduced model is tractable, we are able to analytically obtain the mean contribution of the calcium spike to the somatic membrane potential, while accounting for the background fluctuation (3.4). This analytical form is robust to different levels of fluctuations, whereas first order approximation using linear response theory is not. Beyond the analysis of the contributions of calcium spikes to a neuron's firing activity, this study paves the way to combined theoretical and large-scale numerical investigations of the functional role of calcium spikes and the relationship of correlated synaptic activity to neuronal firing patterns in cortical networks.

## 2. Materials and methods

### 2.1. A first order kinetics model of calcium spike dynamics

The layer 5 pyramidal neuron has previously been represented as a two-compartment point neuron model, in which the calcium spike is modeled using first order kinetics in the distal compartment (Larkum et al., 2004). Here, we take a similar approach, modeling the pyramidal neuron as a system of three connected isopotential compartments, with the somatic compartment [representing the soma and basal dendrites], the proximal [representing the apical dendrite before it bifurcates], and the distal compartment [representing the main bifurcation point (calcium hot-zone) and distal tuft dendrites]. Three compartments instead of two are chosen in order to capture how a hyperpolarizing current at the proximal dendrite can prevent a calcium spike from triggering action potentials at the soma (Larkum et al., 2001). The dynamics of the three-compartment neuron model of the layer 5 pyramidal neuron is described by three coupled first order differential equations governing the time evolution of the membrane potentials of the three compartments

(1) $$\begin{array}{l} \left(\begin{array}{c} C^{\text{d}}{\dot{V}}^{\text{d}}\\ C^{\text{p}}{\dot{V}}^{\text{p}}\\ C^{\text{s}}{\dot{V}}^{\text{s}} \end{array}\right)=(\begin{array}{c} -{\sum^{\text{​}}}_{x\in \{l,e,i\}}g_{x}^{d}(V^{d}-U_{x}^{d})+I_{\text{ca}}\\ -{\sum^{\text{​}}}_{x\in \{l,e,i\}}g_{x}^{p}(V^{p}-U_{x}^{p})+g_{\text{pd}}(\left(V^{\text{d}}-U_{\text{l}}^{\text{d}}\right)\\ -{\sum^{\text{​}}}_{x\in \{l,e,i\}}g_{x}^{s}(V^{s}-U_{x}^{s}) \end{array}\\ \;\begin{array}{c} +g_{\text{pd}}((V^{\text{p}}-U_{\text{l}}^{\text{p}})-(V^{\text{d}}-U_{\text{l}}^{\text{d}}))+I_{\text{AP}}^{\text{d}}\\ -(V^{\text{p}}-U_{\text{l}}^{\text{p}}))+g_{\text{sp}}((V^{\text{s}}-U_{l}^{\text{s}})-(V^{\text{p}}-U_{\text{l}}^{\text{p}}))+I_{\text{AP}}^{\text{p}}\\ +g_{\text{sp}}((V^{\text{p}}-U_{\text{l}}^{\text{p}})-(V^{\text{s}}-U_{l}^{\text{s}})) \end{array}) \end{array}$$

where the superscripts *d*, *p*, and *s* denote the distal, proximal, and somatic compartments, respectively. *C* and *V* refer to capacitance and membrane potential and *g_l_* is the leak conductance. Note that by our choice of the coupling terms between compartment *x* and compartment *y* as $g_{xy}\left(\left(V^{x}-U_{l}^{x}\right)-\left(V^{y}-U_{l}^{y}\right)\right)=g_{xy}\left(V^{x}-V^{y}\right)+g_{xy}\left(U_{l}^{y}-U_{l}^{x}\right)$, there is effectively a constant current $g_{xy}\left(U_{l}^{y}-U_{l}^{x}\right)$ injected into compartment *y*. This is chosen such that the resting potential is equal to $U_{l}^{y}$ when there are no external and calcium currents, $I_{Ap}^{Y}=I_{Ca}=0$, and the excitatory and inhibitory synaptic conductances vanish; *g*_*e*_ = *g*_*i*_ = 0. The synaptic conductances are modeled as alpha functions, i.e., the time course of the conductance evoked by an incoming spike at *t* = 0 is

(2) $$\begin{array}{l} g\left(t\right)=w\frac{e}{\tau_{s}}t\exp\left(-\frac{t}{\tau_{s}}\right), \end{array}$$

where *w* is the strength of the synapse and the maximum amplitude of the alpha function, and τ_*s*_ is the synaptic time constant controlling the rise time of the alpha function. The constants *g*_pd_ and *g*_sp_ are the conductances across the distal-proximal and soma-proximal compartments, and *Ue* and *U*i are the excitatory and inhibitory reversal potentials. We model the calcium current *I*_ca_ using first order kinetics

(3) $$\begin{array}{l} I_{\text{ca}}=g_{\text{ca}}mh\left(U_{\text{ca}}-V^{\text{d}}\right)\\ \tau_{\text{m}}ṁ=m_{\infty }-m\\ \tau_{\text{h}}ḣ=h_{\infty }-h\\ m_{\infty }=\frac{1}{1+\exp\left(m_{\text{slope}}\left(V^{\text{d}}-m_{\text{half}}\right)\right)}\\ h_{\infty }=\frac{1}{1+\exp\left(h_{\text{slope}}\left(V^{\text{d}}-h_{\text{half}}\right)\right)}, \end{array}$$

where *U*ca is the calcium reversal potential, *g*_ca_ the calcium conductance, and *m* and *h* the activating and deactivating functions, respectively, whereby *m*_slope_ > 0 and *h*_slope_ < 0. *m*_∞_ and *h*_∞_ are the respective functions depending on the distal voltage *V*^d^ in a sigmoidal shape, determining the asymptotic values toward which *m* and *h* relax.

The neuron spikes when the somatic membrane potential crosses the adaptive threshold Θ_ad_. At this point the threshold is increased by Θ_+_ from which it relaxes exponentially to Θ_base_ with a time constant of τ_th_. Additionally, a refractory period *t*_ref_ = 2ms is applied to the somatic compartment, during which the somatic leak term *g*^s^_l_ is set to 150nS, as compared to a leak value of 10nS otherwise. This is to emulate the membrane potential coming down to a resting potential value of approximately −60mV from a peak value *V*_peak_ = 30mV to which it jumps upon threshold crossing.

To model the effects of a back-propagating action potential in a three-compartment neuron model, we initialize alpha-shaped currents, with dynamics analogous to the synaptic conductances described above (2), first in the proximal compartment and then in the distal compartment. Specifically, an alpha current *I*^p^_AP_ with maximum amplitude *J*^p^_AP_ and rise time τ^p^_AP_ is initialized in the proximal compartment 1ms after the spike, and a current *I*^d^_AP_ with maximum amplitude *J*^d^_AP_ and rise time τ^d^_AP_ is initialized in the distal compartment 2ms after the spike. The complete parameters of the model are given in Table S1.

### 2.2. Fitting the first order kinetics model

The neuron model is fitted so as to reproduce experimental results illustrated in Figures 1C–E of Larkum et al. (1999a) and Figures 5C2, 6D of Larkum et al. (2001). We perform a parameter scan in a three step procedure. In the first step, we set the spiking threshold to the baseline value Θ_base_, the back-propagating currents *I*^p^_AP_ and *I*^d^_AP_ to 0 and disable the calcium current *I*_ca_. We then fit the leak conductances and capacitance parameters of the neuron model, with search space for capacitance from 50 to 250 pF. Each of the parameters *C*^d^, *C*^p^, and *C*^s^ is being varied independently, as are the leak conductances *g*^d^_l_, *g*^p^_l_, and *g*^s^_l_ from the range 10–50 nS. The fitting criteria are defined by Figure 1C of Larkum et al. (1999a), i.e., a step current stimulus of 1nA for 5ms at the soma compartment initiates an action potential, and also by Figure 5C2 of Larkum et al. (2001) such that, with *I*_ca_ turned off, a hyperpolarizing step current of −0.2nA lasting 50ms at the proximal compartment followed 30ms later by a beta current with time constants 5 and 1 ms of amplitude 2.2nA at the distal compartment does not initiate any action potentials. Only neuron parameter sets (leak conductances and capacitances) that fulfill the above requirements are considered for the next step of fitting.

In step two, *I*_ca_ is enabled and we fit the parameters controlling the calcium dynamics (τ_m_, τ_h_, *m*_half_, *h*_half_, *U*_ca_, and *g*_ca_, with a search space for these parameters of ±20% around those values used in Larkum et al., 2004). The fitting procedure attempts to reproduce (Larkum et al., 1999a) Figures 1D,E, whereby in the first case, a step current of 1 nA is applied at the soma for 5 ms, followed 4 ms later by a beta current with time constants 5 and 1 ms and amplitude 1.1 nA at the distal compartment. This combined stimulus causes a calcium spike and three action potentials. In the second case, a beta current with time constants 5 and 1 ms and amplitude 2.2 nA at the distal compartment causes a calcium spike and two action potentials. In the following we only consider those neuron and calcium parameters that result in the desired response patterns for step three. In this final step, we use the parameter set from step two to fit Θ_ad_ (both the jump amplitude Θ_+_ and time constant τ_th_) and *I*_AP_ amplitudes (τ_AP_ are set to default values of 1 ms) so as to produce the same number of action potentials (three and two, respectively) as in Larkum et al. (1999a) Figures 1D,E. From this step, we finally select the parameter set with minimal values for Θ_ad_ and *I*_AP_ as parameters for the neuron model. This parameter set can be found in Table S1.

### 2.3. Numerical simulations

The numerical simulations are performed with the NEST simulator (Gewaltig and Diesmann, 2007), with a time-step of 0.1ms. The neuron and calcium parameters are listed in the Table S1. In all cases, each compartment of the neuron receives 2000 excitatory synapses and 500 inhibitory synapses, each providing random input at a rate of 1 spikes/s.

In some experiments, coincident inputs are applied to a fraction of the excitatory synapses in the distal compartment according to a multiple interaction process as defined in Kuhn et al. (2003), available in NEST as model “mip_generator.” The coincident events are drawn from a Poissonian mother process with a firing rate ν and copied to each synaptic input independently with a probability *p*. Thus, the contribution of the mother process to each input spike train is a Poisson process with rate ν*p*. In order that every pre-synaptic input has a total firing rate of ν, we apply an independent Poissonian spike train with a firing rate of (1–p) ν to each of the synapses receiving the coincident input, and of firing rate ν to each synapse not receiving coincident input.

## 3. Results

In this section, we first show that a three-compartment model using first order kinetics to obtain the calcium current can reproduce key experimental results and so is an appropriate choice of reference model to evaluate the reduced model developed in this study (Section 3.1). In Section 3.2 we then investigate the response of the model to precise and imprecise synchrony impinging on the distal compartment whilst the neuron receives stochastic input, in order to identify in which regime it is a reasonable approximation to replace the first order kinetics with a fixed waveform triggered by a voltage threshold. We develop this reduced model in Section 3.3 and analyze it in Section 3.4, showing that this simplified calcium dynamics allows us to obtain the voltage excursion at the soma due to a calcium spike analytically.

### 3.1. First order kinetics model reproduces experimental findings

We first investigate whether the three-compartment model with calcium currents modeled using first order kinetics, as described in Section 2.1, is capable of reproducing key experimental phenomena. Using the three step procedure detailed in Section 2.2, we identify a set of parameters for which the neuron model is able to reproduce qualitatively the experimental results presented in Figures 1C–E of Larkum et al. (1999a) and Figures 5C2, 6D of Larkum et al. (2001); the complete set of parameters is given in Table S1.

**Figure 1:**
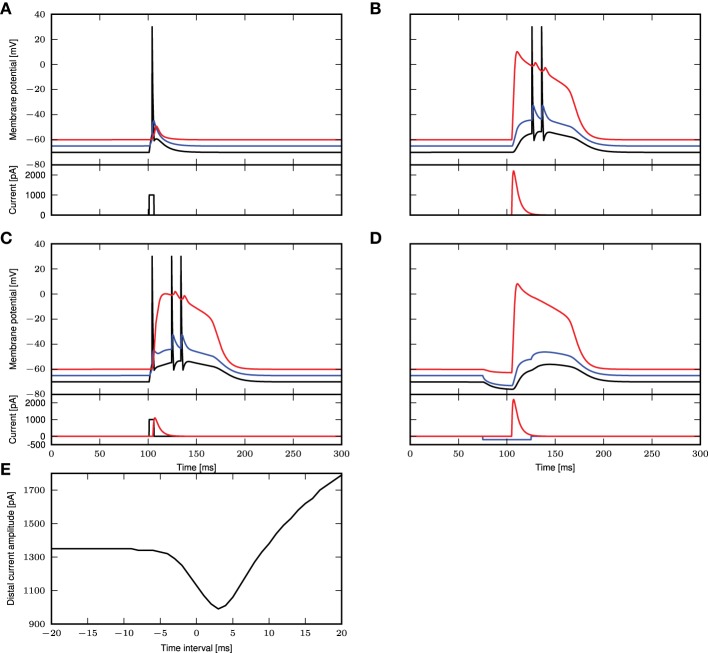
**Comparison of experimental results with simulated results using a three-compartment model with calcium currents modeled using first order kinetics. (A–D)** Top panels show the membrane potentials for each compartment (black: soma, blue: proximal, red: distal), while the lower panels show the corresponding DC stimulation injected at each compartment. See main text for the stimulation details. **(E)** Minimum amplitude of the beta current at the distal compartment required to trigger a calcium spike when applied together with the step current as in **(A)**.

The simulation results for these parameters are shown in Figure 1. In Figure 1A, we reproduce Figure 1C of Larkum et al. (1999a). A step current of 1000pA is applied at the somatic compartment for 5ms, triggering an action potential. In Figure 1B (corresponding to Figure 1E of Larkum et al., 1999a), a beta current, with amplitude 2200pA and time constants 5.0 and 1.0ms, is applied at the distal compartment, triggering a calcium spike which then propagates to the soma and triggers two action potentials. If the same step current is applied again, followed 4ms later by a beta current of half the amplitude, i.e., 1100pA at the distal compartment (see Figure 1D of Larkum et al., 1999a), this triggers a calcium spike at the distal compartment which then causes two additional action potentials, as shown in Figure 1C. If, however, 30ms before applying the beta current, a hyper-polarizing step current of −200pA is applied for 50ms at the proximal compartment (Figure 5C2 of Larkum et al., 2001), the calcium spike is still triggered but is not able to trigger action potentials at the soma, as shown in Figure 1D. A hyper-polarizing current of −200pA as used in Figure 6D of Larkum et al. (2001) is enough to prevent triggering of action potentials by the calcium spike. Figure 1E shows the amplitude of the distal current required to trigger a calcium spike when applied in conjunction with the somatic step current. The time interval refers to time of onset of the distal current relative to the somatic current. This agrees qualitatively with Figure 2D of Larkum et al. (1999a).

These results demonstrate that the three-compartment neuron model with calcium currents modeled by first order kinetics is able to reproduce key experimental results capturing the interaction of calcium spikes with action potentials. We hence conclude that the model is an appropriate choice of reference model against which the reduced model can be evaluated.

### 3.2. Effect of distal calcium currents on the somatic potential

While first order kinetics is able to account for a variety of experimental findings, as shown in the previous section, unfortunately it is not analytically tractable. Consequently, we can only determine the contribution of the calcium current to the somatic membrane potential, and thus the firing behavior of the neuron, by numerical simulation. Ideally, we would like to replace the first order kinetics with something more amenable to further analysis, such as a threshold triggered fixed waveform. We therefore investigate the response of the first order kinetics model to fluctuating input, to determine under what conditions such a simplification would be an appropriate abstraction. In the following we refer to a transient calcium current as a calcium spike, and its contribution to the somatic membrane potential as a calcium somatic potential.

#### 3.2.1. Calcium somatic potential in stochastic input regimes

**Figure 2:**
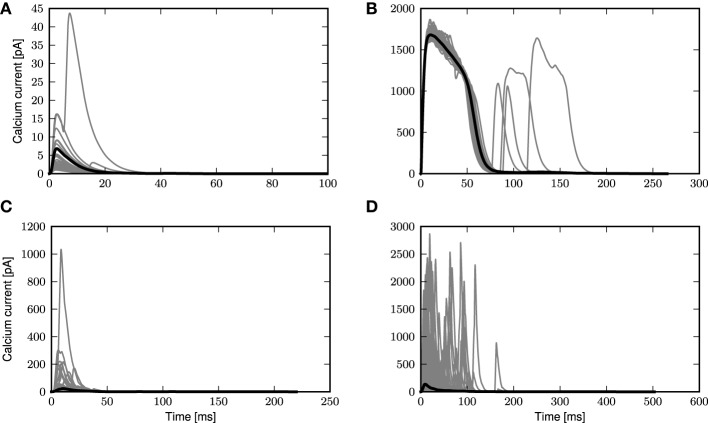
**Calcium spikes generated by stochastic input**. Gray curves are instances of calcium spikes while the black curve is the mean calcium spike for the entire simulation. See main text for details of input. **(A)** Low fluctuation regime with small synchronous inputs. **(B)** Low fluctuation regime with large synchronous events. **(C)** Medium fluctuation inputs with no synchronous events. **(D)** High fluctuation regime with no synchronous events.

We first take a closer look at the dynamics of the three-compartment neuron with first order kinetics receiving noisy input with and without synchronous inputs, as shown in Figure 2. To this end, we apply coincident inputs to the distal dendrite as described in Section 2.3; the input settings for each panel of Figure 2 are given in Table 1. In the weak fluctuating regime, small coincident inputs elicit only small calcium spikes, as shown in Figure 2A. However, with increased probability of coincident inputs as in Figure 2B, a full calcium spike is triggered, which is very consistent in its form across all instances. Calcium spikes elicited not by synchronous inputs but by medium or highly fluctuating inputs as in Figures 2C,D, have much more variable waveforms, even if most calcium spikes are of small amplitudes, with larger ones occurring more frequently in Figure 2D. This is due to the fact that each time a calcium spike is triggered in Figures 2C,D, the underlying excitatory synaptic conductances are noisy and have very different waveforms and amplitudes, unlike in Figures 2A,B.

**Table 1:** **Parameters of stochastic and synchronous input used in Figures 2A–D**.

	**A**	**B**	**C**	**D**
% of distal excitatory synapses receiving correlated inputs	10.0	20.0	0.0	0.0
Copy probability (pair-wise correlation)	0.3	0.5	–	–
Excitatory synaptic weight	0.6nS	0.6nS	5.0nS	12.0nS
Inhibitory synaptic weight	1.0nS	1.0nS	21.2nS	54.4nS

As the calcium spike has been hypothesized as the mechanism by which the layer 5 pyramidal neuron detects coincident inputs (Larkum et al., 1999a, 2009; Spruston, 2008) it is reasonable to assume that it is only triggered by highly coincident inputs, resulting in burst firing. At other times, the neuron should then fire only sparsely given the fluctuating inputs. This is represented by the scenario depicted in Figure 2B, i.e., highly synchronous events with low fluctuating inputs, such that occasional large synchronous inputs trigger calcium spikes, while random synaptic inputs do not. We therefore focus our attention on this scenario for the rest of the manuscript.

**Figure 3:**
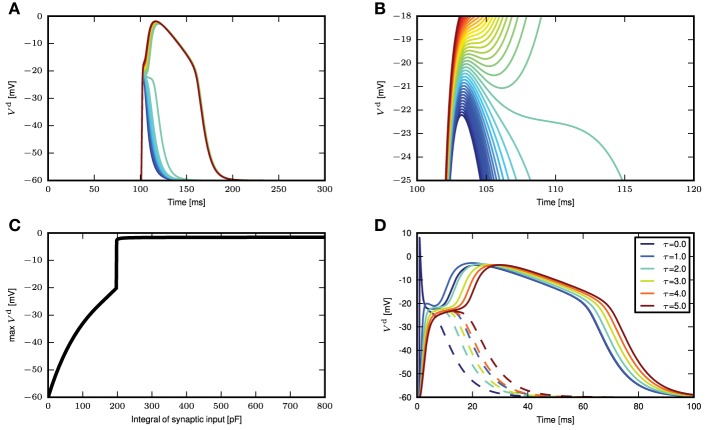
**Response of distal membrane potential to calcium currents triggered by synaptic inputs. (A)** Time course of distal membrane potential for integral of synaptic conductance time course from 180 to 220 pF, illustrated from blue to green to red. **(B)** A zoomed-in view of the bifurcation point in **(A)**. **(C)** The maximum membrane potential reached as a function of the integral of the synaptic conductance time course. **(D)** Distal membrane potential excursions caused by minimal synaptic conductances eliciting a calcium spike (solid curves) and the voltage excursion (dashed curves) due to synaptic conductance excursions reduced by 1 pF. The different colors denote synaptic inputs with different synaptic time constants.

We next stimulate the neuron model with different magnitudes of synchronous input, without the background fluctuations. If the synaptic current has a short time constant compared to the membrane voltage, (Section 2.1) suggests that the amplitude of the membrane voltage deflection mainly depends on the temporal integral of the synaptic conductance. We hence use this parameter to measure the strength of a synaptic input. For small integral conductances Figure 3A shows that the membrane potential quickly relaxes back to the resting level and no calcium current is triggered. Increasing the integral conductance, a full calcium spike is elicited that has a stereotypical form that stays invariant even if the amplitude of the stimulation is further increased. The appearance of a calcium spike can be regarded as a bifurcation. The zoom in into the region of this bifurcation in Figure 3B shows that a full calcium spike is initiated once the membrane potential is above a certain voltage.

Due to the relation of the peak membrane potential to the integral conductance, this threshold-like behavior can alternatively be observed with respect to this input parameter, as shown in Figure 3C. However, the initiation of a calcium spike cannot perfectly be described by a threshold that solely depends on the instantaneous membrane voltage, as shown by the dependence of the calcium waveform on the synaptic time constant in Figure 3D. The reason is that the activating and de-activating functions *m*(*t*) and *h*(*t*) that control the calcium spike are first order differential equations following the sigmoidal functions *m*_∞_(*V*^d^(*t*)) and *h*_∞_(*V*^d^(*t*)), respectively, only with a certain time lag. As a consequence, the initiation of a calcium spike depends to some extent on the time course of *V*^d^(*t*) and as a result there is a small parameter range in which intermediate waveforms appear (see Figure 3B, the voltage excursion in green) that are located between no spike and a full calcium spike. However, given this intermediate parameter range is small, the all or nothing threshold-like behavior prevails. The actual value of the threshold naturally depends on the parameters of the neuron model, such as the membrane capacitance and the leak conductances. These results show that we can approximate the calcium spike with a threshold triggered fixed waveform when in the weak fluctuating regime with occasional large synchronous inputs.

**Figure 4:**
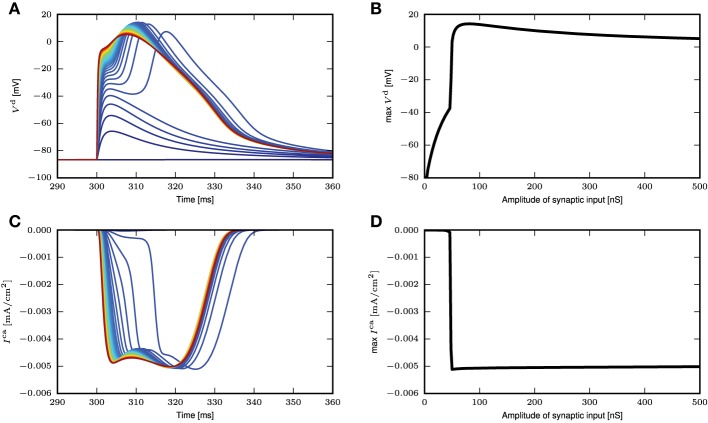
**Response of neuron model from Hay et al. (2011) to synaptic inputs. (A)** Time course of distal membrane potential (650 μm from soma) for amplitude of synaptic conductance from 2 (blue) to 500nS (red), in intervals of 10nS. **(B)** The maximum membrane potential reached as a function of the amplitude of the synaptic conductance. **(C)** Time course of distal calcium current for high voltage activated calcium channels (650 μm from soma). Same color code as in **(A)**. **(D)** The maximum calcium current amplitude reached as a function of the amplitude of the synaptic conductance.

To investigate if the reduction to a threshold-triggered waveform holds true even for full compartmental neuron models such as those in Hay et al. (2011); Almog and Korngreen (2014), we stimulate the neuron model in Hay et al. (2011) with the same simulation settings as Figure 3A. The model is modified such that the sodium channels are deactivated so that the spike generation does not interfere with the membrane potential waveforms at the distal dendrites. An AMPA receptor is then introduced at the calcium hot zone 650 μm from the soma and the local membrane potential and calcium currents from both low and high voltage activated calcium channels are recorded. Figure 4A shows the time course of the local membrane potential when a synaptic input is applied. The voltage waveforms look rather stereotypical whenever a full calcium spike is triggered. The variability, though, is slightly increased compared to the responses shown in Figure 3A. Figure 4B shows the maximum membrane potential reached as a function of the amplitude of the synaptic input. Again a threshold effect is observed and the maximum amplitudes stay relatively constant with increasing synaptic input beyond the threshold value. This constant amplitude is largely due to the reversal potential of excitatory synapses. If a current is used as a stimulus instead of an input from a conductance-based synapse, the threshold effect is still observed, while the membrane potential amplitude continuously, but gradually, increases beyond the threshold. The latter increase is explained by the additional synaptic current stimulus alone; calcium current waveforms and amplitudes are very similar for the increasing current stimuli after the threshold is reached. The corresponding figures for calcium currents and maximum calcium current amplitudes are shown in Figures 4C,D.

#### 3.2.2. Effect of imprecise synchrony on calcium somatic potential

In the previous section we investigated the response of the neuron to precisely synchronous inputs. In particular, we are interested in synchronous inputs with low background fluctuation as in Figure 2B. Using the same settings as in Figure 2B, we now examine whether the approximation of a fixed waveform still holds in a biologically more plausible setting of imprecise synchrony, which would reduce the efficacy of the inputs in eliciting a calcium spike. To this end, we draw the synaptic delays of the inputs mediating the synchronous stimulus to the distal compartment from a Gaussian distribution, truncated at zero.

**Figure 5:**
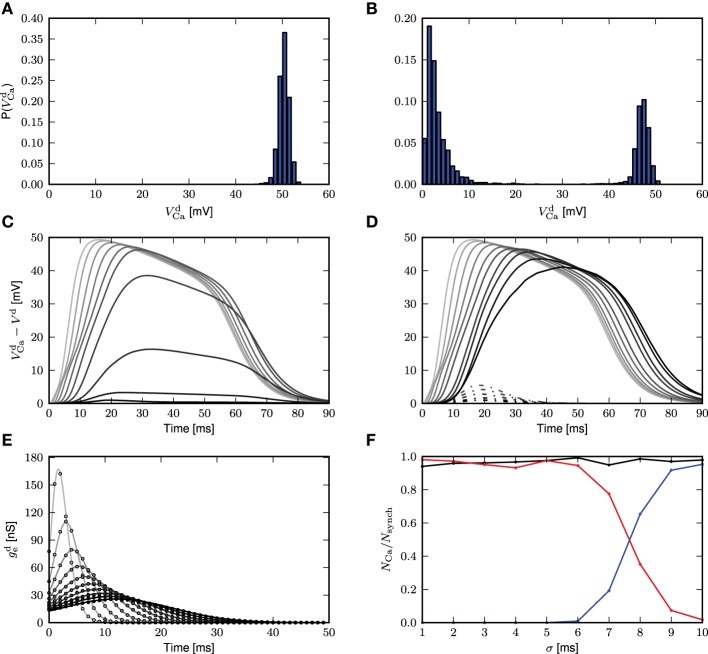
**Calcium spikes elicited by imprecise synchrony**. The delays of the synchronous inputs are drawn from a Gaussian distribution with mean μ = 200 ms and standard deviation σ varying from 1 (light gray) to 10 ms (black). **(A)** Histogram of maximum amplitude of calcium distal potential for σ = 1 ms. **(B)** Histogram of maximum amplitude of calcium distal potential for σ = 8 ms. **(C)** Time course of the mean difference in distal membrane potential following a synchronous input for the three-compartment neuron model with and without calcium currents enabled. The average is taken over all calcium events with difference in membrane potentials exceeding a threshold of 0.001 mV. **(D)** The difference in distal membrane potentials is categorized according to its maximum amplitude being larger (solid curves) or smaller (dashed curves) than 30 mV. The mean time course is then computed within each category separately. **(E)** Time course of the corresponding mean excitatory conductance, analytical results (Equation 4, solid curves) and simulation results (black circles). **(F)** Proportion of synchronous events eliciting calcium currents as a function of the standard deviation of the Gaussian distribution for all amplitudes of calcium distal potential (black), for amplitudes ≥ 30mV (red) and for amplitudes < 30mV (blue).

The histogram of maximum amplitudes of calcium distal potential is shown for σ = 1 ms in Figure 5A, and for σ = 8ms in Figure 5B, whereby both small calcium currents and full calcium spikes are triggered due to the jittered synchronous inputs. The bimodal distribution is a consequence of the threshold effect of the calcium dynamics, leading to an all-or-nothing behavior. We hence define a calcium spike as one that triggers a distal voltage excursion such that $V_{Ca}^{d}-V^{d}\geq 30mV$.

Averaging over all calcium events, Figure 5C demonstrates that the mean distal response to a synchronous stimulus is both delayed and weakened as the standard deviation of the delay distribution increases. The difference in distal membrane potentials between an active and passive neuron model during the time course of calcium spikes are first obtained from the simulation. The time courses are then averaged to compute the mean calcium distal potential. In Figure 5D, the difference in distal membrane potentials are first classified according to their maximum amplitudes, those with maximum amplitudes < 30 mV and those with maximum amplitudes ≥ 30 mV. The mean distal membrane potential is then computed within each class: solid curves for ≥ 30 mV and dashed curves for < 30 mV. For σ ≤ 5 ms, there are no differences in distal membrane potentials with maximum amplitudes < 30 mV.

The reduction of the mean calcium potential shown in Figure 5C is hence mostly a consequence of each dispersed synchronous synaptic conductance triggering a calcium spike with a lower probability. Given that when a full calcium spike is triggered, its amplitude is mainly determined by the intrinsic parameters of the first order kinetics and as shown in Figures 5A,B,D, it is rather insensitive to the dispersion of the incoming pulses. The corresponding mean excitatory conductance for the case of all calcium spikes is shown in Figure 5E, which agrees with its analytical form

(4) $$\begin{array}{l} \langle g_{\text{e}}^{\text{d}}\left(t\right)\rangle =\left[g*𝓝\left(\mu ,\sigma^{2}\right)\right]\left(t\right), \end{array}$$

obtained by convolving the original synaptic conductance waveform with the Gaussian distribution. As the breadth of the delay distribution increases, the conductances are more spread out but their integrals remain the same. As the calcium spike is a threshold triggered event, synaptic inputs having the same integral but spread over a longer time period will be less effective in triggering it. Figure 5F shows the frequency of calcium spikes given the synchronous inputs, whereby all calcium spikes are considered (black), calcium spikes resulting in distal potential with maximum amplitude ≥ 30 mV (red) and calcium spikes resulting in distal potential with maximum amplitude < 30mV. Therefore, with increased jittering, full calcium spikes are triggered with lower frequency, which are also reflected in the mean distal potential (Figures 5C,D). Hence, with imprecise synchrony, the calcium spike can still be approximated by a threshold triggered fixed waveform (as suggested in Figure 5D), although the waveform will have to be adjusted accordingly. How the threshold and waveform may be obtained for the fixed waveform calcium spike is discussed in the following section.

## 3.3. A reduced model of calcium spike dynamics using a fixed waveform with threshold

In the previous sections, we have demonstrated that the calcium spike modeled using first order kinetics can be approximated with a threshold-triggered fixed waveform in the regime of weak fluctuating input with occasional large synchronous events. The waveform is obtained empirically from the mean calcium spike of the neuron model with first order kinetics, which depends on τ_e_ and the background fluctuations, as illustrated in Figure 2B. The neuron model with first order calcium kinetics is embedded in background fluctuations with occasional larger synchronous inputs, with parameters given in Table 1. The calcium current waveform *I*_Ca_ is then obtained from the mean of calcium currents triggered by the large synchronous inputs. The waveform needs to be obtained empirically as it is very sensitive to different τ_e_, as shown in Figure 3D and an analytical treatment seems difficult. What remains to be determined is the value of the effective threshold at which a calcium current is triggered.

### 3.3.1. Determining the calcium spike threshold using voltage slope

**Figure 6:**
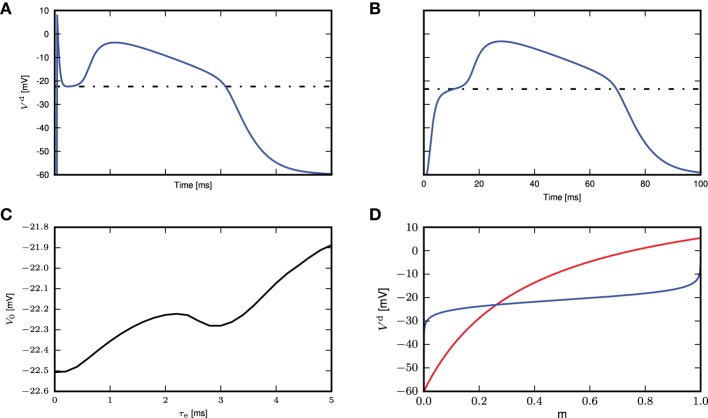
**Determining the calcium spike threshold using voltage slope. (A)** Distal membrane potential excursion due to a calcium spike triggered by a δ pulse synaptic input. Black dashed line denotes the determined threshold value (see text). **(B)** As in **(A)**, but for an alpha synaptic conductance with a time constant τ_e_ = 5 ms. **(C)** Membrane potential *V*_0_ at which the calcium potential slope $\left|\dot{V}\right|$ is minimal as a function of τ_e_. **(D)** Threshold for δ input. The intersection of *V*^d^ (red) against *m* ∈ [0, 1] (numerically obtained from Equation 5) and the inverse function of *m*_∞_(*V*_*d*_) ∈ [0, 1] (blue) against *m* (obtained by numerically inverting *m*_∞_(*V*_*d*_), derived from Equation 3) denotes the theoretical threshold for calcium spikes triggered by δ pulse synaptic inputs.

To determine the calcium threshold, we systematically vary the time constant of the post-synaptic response (we denote by 0 ms the δ impulse, and by 0.2–5.0 ms alpha conductances with the respective time constant) and determine the minimum conductance required to trigger a calcium spike in the first order kinetics model. As the maximum amplitude of a full calcium spike is much higher than that of sub-threshold calcium currents and is effectively independent of input amplitudes and time constants (see Figure 3C), we determine a calcium spike to have been triggered if the calcium current *I*_ca_ exceeds 1100pA. Having established the minimum conductance required to trigger a calcium spike for each synaptic time constant, we then determine the corresponding membrane potential threshold to be that at which $\left|\dot{V}\right|$ is minimal, i.e., the flattest part of the membrane potential trajectory before the calcium spike excursion (Figures 6A,B). The results of this analysis are illustrated in Figure 6C.

We next investigate analytically the emergence of the threshold-like behavior to understand its origin and to find the parameters that determine the threshold voltage. A threshold-like behavior becomes apparent from Figures 3A,B: two EPSP trajectories that lead to either a full calcium spike or not, pass through the same narrow range of voltages with close to vanishing slope $\dot{V}≃0$. If we can analytically obtain the voltage value at which this bifurcation appears, we can compare it to simulation results to check if our analysis captures the essential mechanism underlying this threshold-like behavior. It is sufficient to consider the special case of a δ-shaped current, because the weak dependence of the threshold on the synaptic time constant in Figure 6C suggests that the mechanism can be understood without taking synaptic filtering into account. A δ input causes the membrane potential to instantaneously jump from rest to an elevated level. To determine the threshold, we are hence looking for the voltage levels in all three compartments at which their rate of change vanishes, i.e. where ${\dot{V}}^{d}\;=\;{\dot{V}}^{p}\;=\;{\dot{V}}^{s}\;=\;0$. Hence from (2.1) follows

$$\begin{array}{l} \left(\begin{array}{c} -g_{\text{ca}}mh\left(U_{\text{ca}}-V^{\text{d}}\right)\\ 0\\ 0 \end{array}\right)=\\ \;\left(\begin{array}{ccc} -g_{\text{l}}^{\text{d}}-g_{\text{pd}}g_{\text{pd}}0\\ g_{\text{pd}}-g_{\text{l}}^{\text{p}}-g_{\text{pd}}-g_{\text{sp}} & g_{\text{sp}}\\ 0 & g_{\text{sp}} & -g_{\text{l}}^{\text{s}}-g_{\text{sp}} \end{array}\right)\left(\begin{array}{c} V^{\text{d}}\\ V^{\text{p}}\\ V^{\text{s}} \end{array}\right)\\ \;+\left(\begin{array}{c} g_{\text{l}}^{\text{d}}U_{\text{l}}^{\text{d}}-g_{\text{pd}}U_{\text{l}}^{\text{p}}+g_{\text{pd}}U_{\text{l}}^{\text{d}}\\ g_{\text{l}}^{\text{p}}U_{\text{l}}^{\text{p}}-g_{\text{pd}}U_{\text{l}}^{\text{d}}+g_{\text{pd}}U_{\text{l}}^{\text{p}}-g_{\text{sp}}U_{l}^{\text{s}}+g_{\text{sp}}U_{\text{l}}^{\text{p}}\\ g_{\text{l}}^{\text{s}}U_{l}^{\text{s}}-g_{\text{sp}}U_{\text{l}}^{\text{p}}+g_{\text{sp}}U_{l}^{\text{s}} \end{array}\right). \end{array}$$

Solving for the steady-state voltage, we get

(5) $$\begin{array}{l} {\left(M\right)}^{-1}\left(\begin{array}{c} -g_{\text{ca}}mhU_{\text{ca}}-g_{\text{l}}^{\text{d}}U_{\text{l}}^{\text{d}}+g_{\text{pd}}U_{\text{l}}^{\text{p}}-g_{\text{pd}}U_{\text{l}}^{\text{d}}\\ -g_{\text{l}}^{\text{p}}U_{\text{l}}^{\text{p}}+g_{\text{pd}}U_{\text{l}}^{\text{d}}-g_{\text{pd}}U_{\text{l}}^{\text{p}}+g_{\text{sp}}U_{\text{l}}^{\text{s}}-g_{\text{sp}}U_{l}^{\text{p}}\\ -g_{\text{l}}^{\text{s}}U_{\text{l}}^{\text{s}}+g_{\text{sp}}U_{\text{l}}^{\text{p}}-g_{\text{sp}}U_{l}^{\text{s}} \end{array}\right)=\left(\begin{array}{c} V^{\text{d}}\\ V^{\text{p}}\\ V^{\text{s}} \end{array}\right) \end{array}$$

whereby $M=\left(\begin{array}{ccc} -g_{\text{l}}^{\text{d}}-g_{\text{pd}}-g_{\text{ca}}mh & g_{\text{pd}} & 0\\ g_{\text{pd}} & -g_{\text{l}}^{\text{p}}-g_{\text{pd}}-g_{\text{sp}} & g_{\text{sp}}\\ 0 & g_{\text{sp}} & -g_{\text{l}}^{\text{s}}-g_{\text{sp}} \end{array}\right)$. For a typical set of parameters, τ_m_ ≪ τ_h_, and τ_m_ is close to 0. Consequently, just after the input current we can take an adiabatic approach for *m* and assume *m* = *m*_∞_ and *h* ≃ 1.

We use this steady state fixed point to approximate the calcium spike threshold at the distal compartment. We determine the threshold numerically by plotting *V*^d^ against *m* and the inverse function of *m*_∞_ against *m*, as shown in Figure 6D. The steady state is obtained from the point of intersection, which is −23.1mV, while from simulation, the threshold for δ input is −22.5mV (Figure 6C), which is in quite good agreement, despite the approximations made.

### 3.3.2. Determining the calcium spike threshold using EPSP amplitude

**Figure 7:**
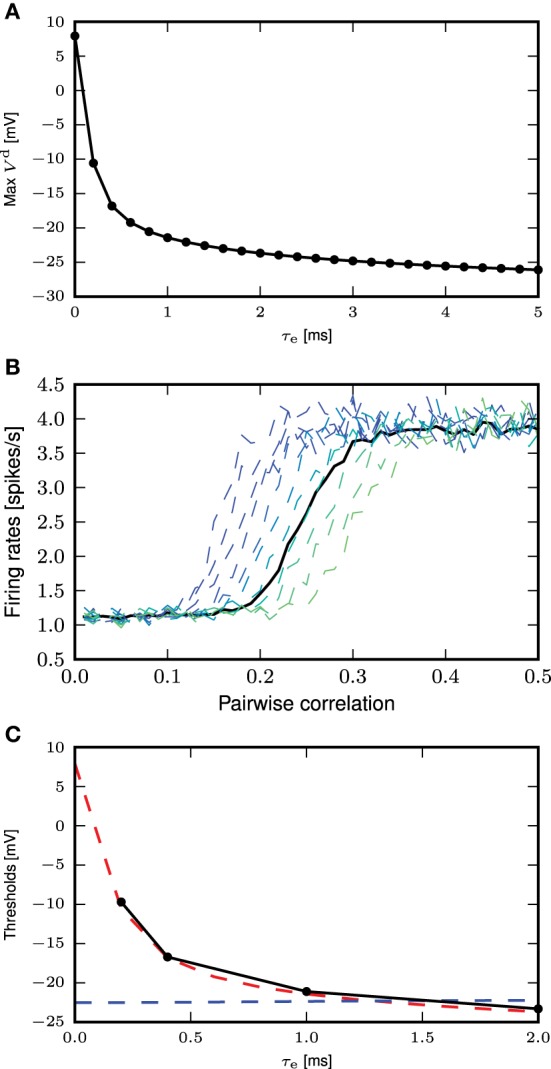
**Determining the calcium spike threshold using EPSP amplitude. (A)** Maximum amplitude of the EPSP (without contribution from calcium spikes) evoked by the minimal conductance required to trigger a full calcium spike as a function of τ_e_. **(B)** Firing rates for the neuron with first order kinetics (solid black curve) and with fixed waveform and different calcium thresholds (dashed curves, from blue to green, −20 to −10mV), against different amount of synchronous inputs with τ_e_ = 0.4ms. **(C)** Comparison of calcium spike threshold obtained by different approaches: results from simulation with background noise (black); derived from the minimum of the calcium potential slope (blue); derived from the maximum EPSP amplitude of the minimal spike triggering conductance (red).

An alternative definition of the voltage threshold is the maximum membrane potential reached by the EPSP for the smallest synaptic input that triggers a full calcium spike (see Figure 7A), without considering the contribution of the calcium spike to the membrane potential. This can be obtained by giving the same stimulus to the neuron models, one with calcium dynamics turned on and another off. The thresholds obtained from the first definition for the different τ_e_ fall within a relatively small range of −21.8 to −22.5 mV (see Figure 6C). As the distal compartment represents the main bifurcation point on the apical dendrite and the distal tuft dendrites, the membrane potential at this location may be highly depolarized and yet have little impact on the soma. In our neuron model, this small impact is manifested in the weak conductances between compartments. In our kinetics calcium model the half activation voltages are *m*_half_ = −21 mV and *h*_half_ = −24mV, values that are close to the thresholds obtained above.

The threshold obtained for the second definition ranges from 8 to −26 mV, with the biggest discrepancy between the two definitions for τ_e_ ≤ 0.5 ms. This can be understood by considering that for small τ_e_, synaptic conductances have to be large so as to allow the first order kinetics of *m* to reach values close to 1. This high conductance is reflected in the EPSP and hence also in the maximum membrane potential. However, this is not reflected in the first definition of a threshold, where $|\dot{V}|\approx 0$.

As it is not *a priori* clear which threshold definition is more appropriate when the neuron model is bombarded with low background fluctuations and occasional large synchronous events, we compared the values derived from the two threshold definitions to values obtained from simulation. To this end, we first determine the firing rate of the neuron model with first order kinetics as a function of the pairwise correlation of synchronous input to the distal compartment, as described in Section 2.3; the parameters of the input spike trains are given in Table 2. We then systematically vary the threshold of the fixed waveform model and likewise determine the firing rate curves. An example of this for τ_e_ = 0.4ms is shown in Figure 7B. The empirical threshold is obtained by finding the best fit of the firing rate curve of the fixed waveform model to the first order kinetics model, as measured by the mean square error (MSE) between the two curves.

**Table 2:** **Parameters of stochastic and synchronous input used in Figures 7B,C**.

# excitatory synapses per compartment	2000
# inhibitory synapses per compartment	500
Rate of Poisson input per synapse (spikes/s)	1.0
Rate of mother process (spikes/s)	1.0
% of distal excitatory synapses receiving inputs from mother process	30.0

Figure 7C shows the values of the threshold obtained from simulation as a function of the time constant of the excitatory synaptic input compared with the values obtained from the two threshold definitions discussed above. For each choice of time constant, the excitatory and inhibitory synaptic weights were adjusted to result in mean somatic membrane potential of −60mV, as detailed in Table 3. These results show that the maximum EPSP amplitude provides a better approximation of empirically obtained calcium threshold for small τ_e_, but for larger values the two approximations give very similar results. This can be understood from the δ input analysis. Firstly, the dynamics of *m* has to be taken into account to obtain the effective threshold, which in the analysis of Section 3.3.1 is assumed to be infinitely fast. Secondly, the EPSP amplitude that just triggers a calcium spike, implicitly contains the temporal dynamics of *m* to some extent, as the membrane potential first relaxes before the calcium spike sets in [namely while *m*(*t*) approaches $m_{\infty }\left(V^{d}\right)$ on the time scale τ_m_]. This effect is best observed in the case of small τ_e_.

**Table 3:** **Synaptic configurations leading to a mean somatic membrane potential of −60 mV in Figure 7C**.

Time constant of excitatory synapses τ_e_ (ms)	0.2	0.4	1.0	2.0
Excitatory synaptic weight (nS)	2.9	1.5	0.6	0.4
Inhibitory synaptic weight (nS)	1.0	1.0	1.0	2.2

A similar method of comparison using the number of calcium spikes triggered instead of the firing rate yielded the same results (data not shown). This can also be intuitively understood by considering that in the low fluctuating regime with large synchronous inputs, calcium spikes are only triggered by sufficiently large synchronous events. This means that synchronous inputs must result in an EPSP of a certain minimum amplitude for a calcium spike to be triggered. In a low fluctuating regime, this minimum amplitude is close to the EPSP amplitude of the minimal calcium spike triggering conductance, i.e., the second definition of the threshold. We therefore conclude that the definition based on the maximum EPSP amplitude is more appropriate for our activity regime of interest.

**Figure 8:**
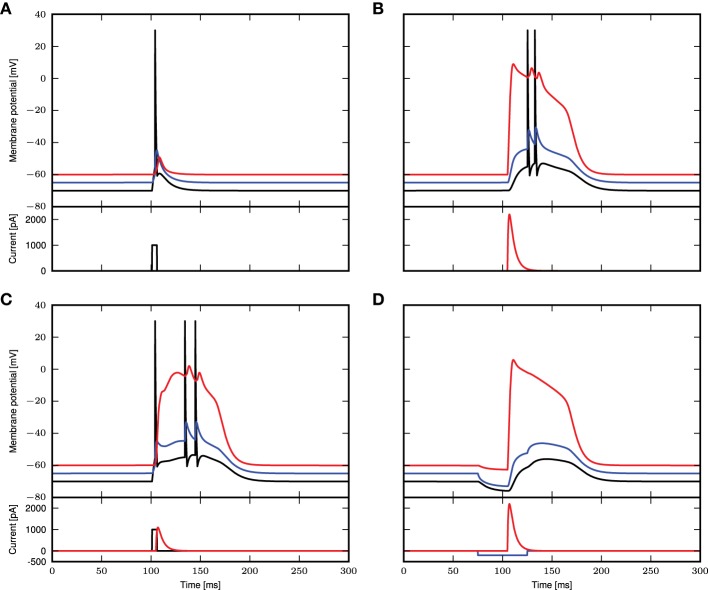
**Simulation results using a threshold and fixed waveform for the calcium spike, simulation settings as in Figure 1. (A–D)** Top panels show the membrane potentials for each compartment (black: soma, blue: proximal, red: distal), while the lower panels show the corresponding DC stimulation injected at each compartment.

Next, we obtain a calcium spike threshold of −25mV (as defined by maximum EPSP amplitude following a beta-shaped current stimulus to the distal compartment with time constants of 5.0 and 1.0ms) and the corresponding waveform of the calcium current. We use the threshold and the waveform to set up the simplified calcium dynamics, thus effectively removing the eight dynamic variables required for the first order kinetics to obtain a reduced three-compartment neuron model. All other neuron parameters remain unchanged. We then assess the ability of the reduced model to reproduce key experimental findings, using the same stimuli and protocols described in 3.1 and Figure 1, corresponding to Figures 1C–E of Larkum et al. (1999a) and Figures 5C2, 6D of Larkum et al. (2001). Figure 8 demonstrates that despite the substantial reduction in model complexity, the threshold triggered fixed waveform neuron model reproduces all the experimental results just as accurately as the first order kinetics model, with no further parameter tuning required.

## 3.4. Contribution of the calcium potential to the somatic potential

In the previous sections, we showed how to arrive at the threshold and waveform in the simplified model. In this section we first use the simplified model to derive the analytical form of the somatic membrane potential excursion due to a calcium spike treating the calcium current as a small perturbation to linear order. As it turns out that this result only agrees well with simulation when background fluctuations are small, in Section 3.4.2 and Section 3.4.3 we derive two alternative forms which also agree well in the case of large background fluctuations.

### 3.4.1. Approximating the calcium potential using first order approximation

Using the empirical values of the mean calcium spike obtained earlier from Figure 2B as the fixed waveform for the simplified calcium dynamics, the calcium potential in the fluctuation regime can be linearly approximated (Papoulis, 1991; Kuhn et al., 2004). The dynamics of the neuron model in the steady state can be expressed using mean conductances and mean potential. Rearranging (1) in the steady state and for *I*^p^_AP_ = *I*^d^_AP_ = 0 and without the contribution due to the calcium spike *I*_ca_ = 0 we get with the definition of the 3 by 3 matrix

(6) $$\begin{array}{l} M=\;(\begin{array}{c} -(g_{\text{l}}^{\text{d}}+\langle g_{\text{e}}^{\text{d}}\rangle +\langle g_{\text{i}}^{\text{d}}\rangle +g_{\text{pd}})∕C^{\text{d}}\\ g_{\text{pd}}∕C^{\text{p}}\\ 0 \end{array}\\ \;\begin{array}{c} g_{\text{pd}}∕C^{\text{d}}\\ -(g_{\text{l}}^{\text{p}}+\langle g_{\text{e}}^{\text{p}}\rangle +\langle g_{\text{i}}^{\text{p}}\rangle +g_{\text{pd}}+g_{\text{sp}})∕C^{\text{p}}\\ g_{\text{sp}}∕C^{\text{s}} \end{array}\\ \;\begin{array}{c} 0\\ g_{\text{sp}}∕C^{\text{p}}\\ -(g_{\text{l}}^{\text{s}}+\langle g_{\text{e}}^{\text{s}}\rangle +\langle g_{\text{i}}^{\text{s}}\rangle +g_{\text{sp}})∕C^{\text{s}} \end{array}) \end{array}$$

the time-averaged equation

$$\begin{array}{l} -M\left(\begin{array}{c} \langle V^{\text{d}}\rangle \\ \langle V^{\text{p}}\rangle \\ \langle V^{\text{s}}\rangle \end{array}\right)=\;(\begin{array}{c} (g_{\text{l}}^{\text{d}}U_{\text{l}}^{\text{d}}+\langle g_{\text{e}}^{\text{d}}\rangle U_{\text{e}}^{\text{d}}+\langle g_{\text{i}}^{\text{d}}\rangle U_{\text{i}}^{\text{d}}\\ (g_{\text{l}}^{\text{p}}U_{\text{l}}^{\text{p}}+\langle g_{\text{e}}^{\text{p}}\rangle U_{\text{e}}^{\text{p}}+\langle g_{\text{i}}^{\text{p}}\rangle U_{\text{i}}^{\text{p}}\\ (g_{\text{l}}^{\text{s}}U_{\text{l}}^{s}+\langle g_{\text{e}}^{\text{s}}\rangle U_{\text{e}}^{\text{s}}+\langle g_{\text{i}}^{\text{s}}\rangle U_{\text{i}}^{\text{s}} \end{array}\\ \;\begin{array}{c} +g_{\text{pd}}U_{\text{l}}^{\text{d}}-U_{\text{l}}^{\text{p}})∕C^{\text{d}}\\ +g_{\text{pd}}U_{\text{l}}^{\text{p}}-U_{\text{l}}^{\text{d}}+g_{\text{sp}}U_{\text{l}}^{\text{p}}-U_{l}^{s})∕C^{\text{p}}\\ +g_{\text{sp}}U_{l}^{s}-U_{\text{l}}^{\text{p}})∕C^{\text{s}}, \end{array}) \end{array}$$

from which we determine the stationary voltage 〈*V*^*x*^〉. If the system is perturbed with a calcium spike at the distal compartment, we can write the perturbed solution as a small deflection ϵ from the stationary state 〈*V*〉, such that with

(7) $$\begin{array}{ll} \left(\begin{array}{c} V^{\text{d}}\left(t\right)\\ V^{\text{p}}\left(t\right)\\ V^{\text{s}}\left(t\right) \end{array}\right) & =\left(\begin{array}{c} \langle V^{\text{d}}\rangle +\epsilon^{\text{d}}\left(t\right)\\ \langle V^{\text{p}}\rangle +\epsilon^{\text{p}}\left(t\right)\\ \langle V^{\text{s}}\rangle +\epsilon^{\text{s}}\left(t\right) \end{array}\right) \end{array}$$

we obtain a set of linear differential equations for the deviations ϵ. The contribution of the calcium spike to the free membrane potential of each compartment can be expressed as

(8) $$\begin{array}{l} \left(\begin{array}{l} V^{\text{d}}\left(t\right)\\ V^{\text{p}}\left(t\right)\\ V^{\text{s}}\left(t\right) \end{array}\right)=\left(\begin{array}{l} \langle V^{\text{d}}\rangle \\ \langle V^{\text{p}}\rangle \\ \langle V^{\text{s}}\rangle \end{array}\right)+\overset{t}{\underset{0}{\int }}\exp\left(\left(t-s\right)M\right)\left(\begin{array}{c} I_{\text{ca}}\left(s\right)/C^{\text{d}}\\ 0\\ 0 \end{array}\right)ds\\ =\int_{0}^{t}\left(\begin{array}{c} \phi_{1}\\ \phi_{2}\\ \phi_{3} \end{array}\right)\left(t-s\right)I_{\text{ca}}\left(s\right)∕C^{\text{d}}ds, \end{array}$$

whereby *I*_ca_ refers to the calcium fixed waveform and the functions ϕ_1, …, 3_ can in principle be obtained as the matrix exponential in Equation (8).

**Figure 9:**
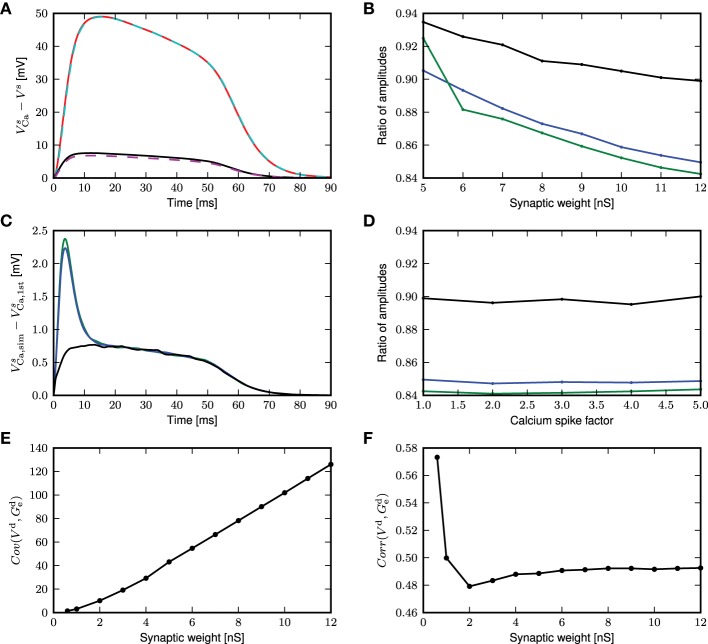
**First order approximation of calcium somatic potential. (A)** The mean calcium somatic potential evoked by randomly triggered calcium spikes obtained from simulation (solid: red, black) and first order approximation (dashed: cyan, magenta) for synaptic weight 0.6, 12nS, respectively. **(B)** Ratio of the maximum amplitude of first order approximation to that of simulated calcium somatic potential as a function of excitatory synaptic weights; for calcium spikes triggered randomly (black), using a threshold of −30 mV (blue), and using a threshold of −21mV (green). **(C)** Difference in calcium somatic potential between simulation results and first order approximation; colors as in **(B)**. **(D)** Ratio of the maximum amplitude of first order approximation to that of simulated calcium somatic potential as a function of multiplicative factor of calcium spike waveform; colors as in **(B)**. **(E)** Covariance of distal membrane potential *V*^d^ and excitatory conductances *g*^d^_e_ as a function of excitatory synaptic weights. **(F)** Correlation coefficients of distal membrane potential *V*^d^ and excitatory conductances *g*^d^_e_ as a function of excitatory synaptic weights.

However, this first order approximation works well for small fluctuating inputs and underestimates the calcium potential under large fluctuating inputs, as shown in Figure 9A. The mean calcium potential is obtained from the mean difference in somatic membrane potential between two neurons receiving exactly the same synaptic inputs, one with and one without calcium currents. We investigate the dependence of this effect on the strength of the excitatory weights in Figure 9B. In order to have comparable results, we analytically derive the corresponding inhibitory weights so as to maintain a mean free membrane potential of −60mV at the soma. The discrepancy between the theoretical prediction and the simulated result becomes more pronounced at larger synaptic weights, due to an increase in the correlation of the membrane potential and conductances. The deviation is even greater if the calcium spike is elicited at thresholds above mean distal membrane potential, as there is a jump at the onset of the calcium potential (see Figure 9C).

The deviation of the theoretical approximation from simulated results are hardly affected by the amplitude of the calcium spike (see Figure 9D), and reversal potential (results not shown). This can be understood by considering that the differential equation in this approximation (Equation 8) is linear, so the contribution ϵ to the membrane potential due to the calcium current is additive and fulfills

(9) $$\begin{array}{l} C\dot{\epsilon }\left(t\right)=-g_{\text{l}}\epsilon \left(t\right)-g_{\text{e}}\left(t\right)\epsilon \left(t\right)-g_{\text{i}}\left(t\right)\epsilon \left(t\right)+I_{\text{ca}}\left(t\right). \end{array}$$

From (9), we observe that synaptic conductances act as multiplicative noise on the voltage contribution of a calcium spike. The covariance between membrane potential and excitatory conductance increases with synaptic weight, as shown in Figure 9E. The average effect of—for example—the excitatory driving force is 〈*g*_e_(*t*)*V*(*t*)〉 = 〈*g*_e_(*t*)〉〈*V*(*t*)〉 + Cov(*g*_e_, *V*), hence it contains the term from first order approximation, 〈*V*(*t*)〉〈*g*_*e*_(*t*)〉, and in addition the covariance between the voltage and the conductance. Neglecting the covariance explains why the first order approximation begins to deviate from simulation results as the fluctuating inputs get larger. The magnitude of the effect of the excitatory conductance depends on the amplitude of fluctuations in the membrane potential and excitatory conductances and on their co-fluctuations as shown by Cov(*g*_e_, *V*) = Corr(*g*_e_, *V*)σ(*g*_e_)σ(*V*), where Corr is Pearson's correlation coefficient. Hence, while the correlation coefficient between membrane potential and conductances decreases with synaptic weights (see Figure 9F), the larger fluctuations in membrane potential and conductances more than make up for it. The decrease in correlation coefficient can be explained by the fact that with larger conductances, the membrane potential approaches the reversal potential *U*_e_, effectively reducing the driving force and therefore the contribution of excitatory conductances to the membrane potential. In the following, we derive alternative forms for the excursion of the somatic membrane potential following a calcium spike to improve the fit to the simulated results for large fluctuating inputs.

### 3.4.2. Accounting for the deviation semi-analytically

Accounting for the correlations between membrane potential and fluctuating synaptic inputs, we arrive at a better theoretical approximation. To illustrate the main effect, it is sufficient to consider a single compartment and a δ-shaped calcium current *I*_ca_ = *A*δ(*t*), where *A* is the pre-factor determining the amplitude. Integrating (9) from *t* = 0 to *t* = ∞, we get

(10) $$\begin{array}{l} \underset{{}_{\epsilon (\infty )-\epsilon (0)=0}}{\underset{︸}{\int_{0}^{\infty }\dot{\epsilon }(t)dt}}=-\frac{g_{\text{l}}}{C}\int_{0}^{\infty }\epsilon (t)dt\\ \;-\frac{1}{C}\int_{0}^{\infty }(g_{\text{e}}(t)+g_{\text{i}}(t))\epsilon (t)dt+\frac{\text{A}}{\text{C}}, \end{array}$$

so with *g*_*ei*_(*t*) = *g*_*e*_(*t*) + *g*_*i*_(*t*)

$$\begin{array}{lll} \overset{\infty }{\underset{0}{\int }}g_{\text{ei}}\left(t\right)\epsilon \left(t\right)dt & = & A-g_{\text{l}}\overset{\infty }{\underset{0}{\int }}\epsilon \left(t\right)dt \end{array}$$

we get an equation relating the effective driving force due to the conductance to the integral of the post-synaptic potential. From observation in simulation, the calcium potential decays exponentially. We therefore assume that it follows an effective differential equation of the form

$$\begin{array}{l} C{\dot{V}}_{\text{eff}}=-g_{\text{l}}V_{\text{eff}}-g_{\text{eff}}V_{\text{eff}}+I_{\text{ca}}, \end{array}$$

where the effective conductance *g*_eff_ is a constant to be determined. Hence, integrating the latter equation as above, we get

$$\begin{array}{l} \overset{\infty }{\underset{0}{\int }}g_{\text{eff}}V_{\text{eff}}\left(t\right)dt=A-g_{\text{l}}\overset{\infty }{\underset{0}{\int }}V_{\text{eff}}\left(t\right)dt, \end{array}$$

which, by re-arranging, yields an expression for the effective leak

$$\begin{array}{l} \overset{\infty }{\underset{0}{\int }}V_{\text{eff}}\left(t\right)dt=\frac{A}{g_{\text{eff}}+g_{\text{l}}}. \end{array}$$

By assuming that the driving force can be expressed by the effective conductance $g_{\text{eff}}\int_{0}^{\infty }V_{\text{eff}}\left(t\right)dt=\int_{0}^{\infty }g_{\text{ei}}\left(t\right)\;\epsilon \;\left(t\right)dt$,

(11) $$\begin{array}{l} g_{\text{eff}}=\frac{A}{\int_{\infty }^{0}\epsilon (t)dt}-g_{\text{l}}. \end{array}$$

The calculation above shows that the effective leak of the neuron model in the fluctuation regime (where conductances and membrane potential are correlated) can be corrected by considering the integral of the calcium potential. This correction is semi-analytic as we need to determine the calcium potential through simulation to obtain a better theoretical approximation. For our three compartment model we obtain from Equation (1) the coupled set of first order differential equations

(12) $$\begin{array}{ll} \left(\begin{array}{c} C^{d}\dot{\epsilon^{\text{d}}}\left(t\right)\\ C^{p}\dot{\epsilon^{\text{p}}}\left(t\right)\\ C^{s}\dot{\epsilon^{\text{s}}}\left(t\right) \end{array}\right) & =-\left(M_{0}+M_{1}\left(t\right)\right)\left(\begin{array}{c} \epsilon^{\text{d}}\left(t\right)\\ \epsilon^{\text{p}}\left(t\right)\\ \epsilon^{\text{s}}\left(t\right) \end{array}\right)+\left(\begin{array}{c} A\delta \left(t\right)\\ 0\\ 0 \end{array}\right),\; \end{array}$$

where we defined the time-independent matrix

$$\begin{array}{lll} M_{0} & = & \left(\begin{array}{ccc} g_{\text{l}}^{\text{d}}+g_{\text{pd}} & -g_{\text{pd}} & 0\\ -g_{\text{pd}} & g_{\text{l}}^{\text{p}}+g_{\text{pd}}+g_{\text{sp}} & -g_{\text{sp}}\\ 0 & -g_{\text{sp}} & g_{\text{l}}^{\text{s}}+g_{\text{sp}} \end{array}\right) \end{array}$$

and the time dependent matrix

$$\begin{array}{lll} M_{1}\left(t\right) & = & \left(\begin{array}{ccc} g_{e}^{d}\left(t\right)+g_{i}^{d}\left(t\right) & 0 & 0\\ 0 & g_{e}^{p}\left(t\right)+g_{i}^{p}\left(t\right) & 0\\ 0 & 0 & g_{e}^{s}\left(t\right)+g_{i}^{s}\left(t\right) \end{array}\right). \end{array}$$

Integrating both sides of Equation (12) from *t* = 0 to *t* = ∞ as before we obtain

$$\overset{\infty }{\underset{0}{\int }}M_{1}\left(t\right)\left(\begin{array}{c} \epsilon^{\text{d}}\left(t\right)\\ \epsilon^{\text{p}}\left(t\right)\\ \epsilon^{\text{s}}\left(t\right) \end{array}\right)dt=\left(\begin{array}{c} A\\ 0\\ 0 \end{array}\right)-M_{0}\overset{\infty }{\underset{0}{\int }}\left(\begin{array}{c} \epsilon^{\text{d}}\left(t\right)\\ \epsilon^{\text{p}}\left(t\right)\\ \epsilon^{\text{s}}\left(t\right) \end{array}\right)dt.$$

Replacing the left hand side by an effective term, $\int_{0}^{\infty }M_{1}\left(t\right)\left(\begin{array}{c} \epsilon^{d}\left(t\right)\\ \epsilon^{p}\left(t\right)\\ \epsilon^{s}\left(t\right) \end{array}\right)dt≃\int_{0}^{\infty }\left(\begin{array}{c} g_{\text{eff}}^{\text{d}}\epsilon^{d}\left(t\right)\\ g_{\text{eff}}^{\text{p}}\epsilon^{p}\left(t\right)\\ g_{\text{eff}}^{\text{s}}\epsilon^{s}\left(t\right) \end{array}\right)dt,$ where we assume that the effective conductance mainly depends on the voltage of the corresponding compartment, we get the effective conductances for a three-compartment neuron model with a calcium spike of any fixed waveform as

$$\begin{array}{l} \left(\begin{array}{c} g_{\text{eff}}^{\text{d}}\\ g_{\text{eff}}^{\text{p}}\\ g_{\text{eff}}^{\text{s}} \end{array}\right)=\left(\begin{array}{ccc} (\int_{0}^{\infty }\epsilon^{\text{d}}(t)dt{)}^{-1} & 0 & 0\\ 0 & (\int_{0}^{\infty }\epsilon^{\text{p}}(t)dt{)}^{-1} & 0\\ 0 & 0 & (\int_{0}^{\infty }\epsilon^{\text{s}}(t)dt{)}^{-1} \end{array}\right)\\ \;\left(\left(\begin{array}{c} A\\ 0\\ 0 \end{array}\right)-M_{0}\overset{\infty }{\underset{0}{\int^{\text{​}}}}\left(\begin{array}{c} \epsilon^{\text{d}}(t)\\ \epsilon^{\text{p}}(t)\\ \epsilon^{\text{s}}(t) \end{array}\right)dt\right), \end{array}$$

where *A* refers to the integral of the calcium current time course. The values for *g*_eff_ obtained above can then be used in place of the respective 〈*g*_e_〉 + 〈*g*_i_〉 in (8) to obtain the corrected calcium potential approximation. If we assume that the conductances in Equation (10) can be sufficiently well-approximated by their mean, then the above consideration yields

$$\begin{array}{l} g_{\text{eff}}=\langle g_{\text{e}}\rangle +\langle g_{\text{i}}\rangle , \end{array}$$

which is the same as our first order approximation presented above.

### 3.4.3. Accounting for the deviation analytically

The semi-analytical approach requires the integral of the calcium potential for approximating the potential waveform, which must be obtained empirically. We now develop a purely analytical approach capable of approximating the waveform of the calcium potential. From Richardson and Gerstner (2005), the mean membrane potential for a single compartment neuron model in the fluctuation regime can be corrected as

$$\begin{array}{l} \langle V\rangle =E_{0}-\frac{\sigma_{\text{e}}^{2}}{G_{0}^{2}}\left(\langle g_{\text{e}}\rangle -E_{0}\right)\frac{\tau_{\text{e}}}{\tau_{\text{e}}+\tau_{0}}-\frac{\sigma_{i}^{2}}{G_{0}^{2}}\left(\langle g_{\text{i}}\rangle -E_{0}\right)\frac{\tau_{\text{i}}}{\tau_{\text{i}}+\tau_{0}}, \end{array}$$

where *G*_0_ = *g*_l_ + 〈*g*_e_〉 + 〈*g*_i_〉, $\tau 0=\frac{C}{G0}$, τ_e_ and τ_i_ are the excitatory and inhibitory synaptic time constants. $E_{0}=\frac{g_{l}U_{l}+\langle g_{e}\rangle U_{e}+\langle g_{i}\rangle U_{i}}{G_{0}}$ is the mean membrane potential from the first order approximation and σ^2^_e_ and σ^2^_i_ are the variances of the excitatory and inhibitory conductances. Rearranging, we obtain

(13) $$\begin{array}{l} G_{0}\langle V\rangle =g_{\text{l}}U_{\text{l}}+\langle g_{\text{e}}\rangle U_{\text{e}}+\langle g_{\text{i}}\rangle Ui-\\ \frac{\sigma_{\text{e}}^{2}}{G_{0}}\left(\langle g_{\text{e}}\rangle -E_{0}\right)\frac{\tau_{\text{e}}}{\tau_{\text{e}}+\tau_{0}}-\frac{\sigma_{\text{i}}^{2}}{G_{0}}\left(\langle g_{\text{i}}\rangle -E_{0}\right)\frac{\tau_{\text{i}}}{\tau_{\text{i}}+\tau_{0}}. \end{array}$$

As we are interested in a small deviation of the membrane potential from the value *E*_0_ resulting from static conductances, 〈*V*〉 = *E*_0_ + δ*V*, we can let *E*_0_≃ 〈V〉. Substituting and rearranging (13), we get

$$\begin{array}{l} 0≃g_{\text{l}}\left(U_{\text{l}}-\langle V\rangle \right)+\left(\langle g_{\text{e}}\rangle -\frac{\sigma_{\text{e}}^{2}}{G_{0}}\frac{\tau_{\text{e}}}{\tau_{\text{e}}+\tau_{0}}\right)\left(U_{\text{e}}-\langle V\rangle \right)\\ +\left(\langle g_{\text{i}}\rangle -\frac{\sigma_{\text{i}}^{2}}{G_{0}}\frac{\tau_{\text{i}}}{\tau_{\text{i}}+\tau_{0}}\right)\left(Ui-\langle V\rangle \right) \end{array}$$

Hence the effective conductance follows as the factors multiplying the mean membrane potential

$$\begin{array}{l} g_{\text{eff}}=\left(\langle g_{\text{e}}\rangle -\frac{\sigma_{\text{e}}^{2}}{G_{0}}\frac{\tau_{\text{e}}}{\tau_{\text{e}}+\tau_{0}}\right)+\left(\langle g_{\text{i}}\rangle -\frac{\sigma_{\text{i}}^{2}}{G_{0}}\frac{\tau_{\text{i}}}{\tau_{\text{i}}+\tau_{0}}\right). \end{array}$$

The analytically obtained *g*_eff_ for a single compartment can be applied analogously to each of the three compartments in our neuron model so as to obtain $g_{e\mathrm{ff}}^{x}$ for *x* ∈ {*s, p, d*}.

**Figure 10:**
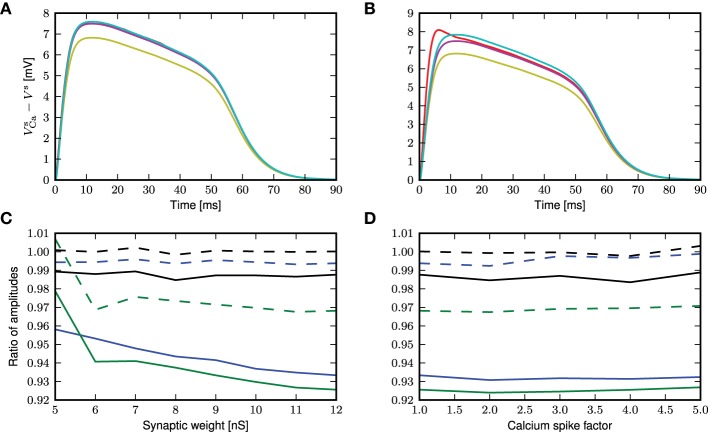
**Comparison of different approaches to account for multiplicative synaptic noise. (A)** The mean calcium somatic potential evoked by randomly triggered calcium spikes obtained from simulation (red curve) and derived from first order approximation (yellow), semi-analytical (cyan), analytical (magenta) approaches. **(B)** As in **(A)** for calcium spike triggered at a threshold of −21 mV. **(C)** Ratio of maximum amplitude of calcium potential for analytical (solid curves) and semi-analytical (dashed curves) approximations to the simulated results as a function of the excitatory synaptic weight, for randomly activated calcium spikes (black) and for voltage-triggered calcium spikes with a threshold of −30 mV (blue) and −21 mV (green). **(D)** As in **(C)** but as a function of the multiplicative factor of calcium spike waveform.

The semi-analytical and analytical results are shown in Figure 10. In the case of a randomly triggered calcium spike, both approaches give the same approximation (see Figure 10A), whereas in the case of a threshold-triggered calcium spike, the two approaches give slightly different results, but still perform better than first order approximation (see Figure 10B). The semi-analytical approach slightly over-estimates, while the analytical result is very close to the simulation result after the initial jump that is exhibited by the simulated results (Figure 10B). If we consider the ratio of peak amplitudes (as shown in Figure 10C), this initial jump in the onset of the calcium potential causes both approaches to slightly underestimate the simulation results. The initial jump increases for higher calcium thresholds and synaptic weights, exacerbating this effect. As before, the deviations of the theoretical approximations from simulated results are hardly affected by the amplitude of the calcium spike (see figure 10D).

**Figure 11:**
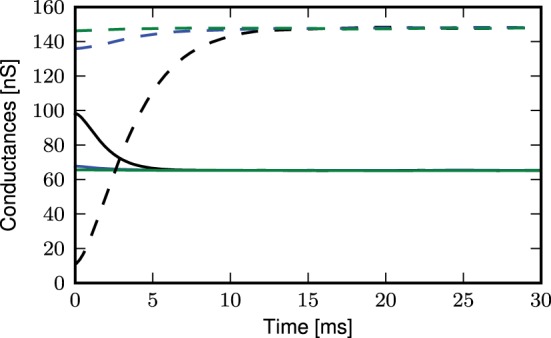
**Mean excitatory (solid curves) and inhibitory (dashed curves) conductances during an average calcium spike triggered by threshold of −21 mV: distal (black), proximal (blue), somatic (green)**.

The deviation of the theoretical predictions from the simulation results with threshold-triggered calcium spikes can be explained by the fact that when a calcium spike is threshold-triggered, the mean excitatory and inhibitory conductances deviate from their stationary values as shown in Figure 11: the excitatory conductances are higher than the stationary value indicated by the convergence of the curves, the inhibitory conductance is lower. Both conductances relax back to their respective stationary means on the time scales of their respective synaptic time constants. With a lower net mean conductance 〈*g*_e_(*t*)〉 + 〈*g*_i_(*t*)〉 at the onset of the calcium spike, the initial calcium potential from simulation is larger than the analytical results (Figure 9C). This is however not the case for randomly triggered calcium spikes, where the mean synaptic conductances stay the same throughout (results not shown).

The semi-analytical result makes use of the integral of the calcium potential to obtain an effective leak conductance *g*_eff_, which is a time average considering the whole time course of the calcium potential. Hence, the simulated and semi-analytically obtained calcium potential have the same integral, by construction. Consequently, the jump at the onset of the calcium potential is averaged over the entire time course of the calcium potential, resulting in an overall slight over-estimation of the simulation results as shown in (Figure 10B). Thus, the analytical approximation agrees with simulation other than during the onset of the calcium potential, while the semi-analytical approximation generally over-estimates the simulated calcium potential.

## 4. Discussion

In this study we have shown that a three compartment neuron model with calcium dynamics modeled using first order kinetics is able to reproduce a variety of experimental findings gathered using *in-vitro* protocols (Larkum et al., 1999a, 2001). In general, the generated calcium spike has neither a fixed waveform nor a threshold (see Figure 2). However, we have shown that when the calcium spike is triggered by large synchronous inputs in the regime of low background fluctuations, it does so in a threshold-like manner, and has a stereotypical waveform (see Figure 3) that depends on the given synaptic time constant τ_e_. Using analysis and simulation, we extracted the parameters of the threshold and waveform from the first order kinetics model. The threshold depends on the time constant τ_m_ of the calcium activating function as well as the membrane time constant. It turns out that the threshold that agrees best with simulation is empirically obtained from the maximum EPSP amplitude (without contribution from calcium spikes) evoked by the minimal conductance required to trigger a full calcium spike (see Figure 7). With both the threshold and waveform known, the calcium spike can be modeled with the analytically tractable threshold-triggered fixed waveform. We use threshold and waveform to define a threshold-triggered fixed waveform model of the calcium spike. The number of calcium parameters are hereby considerably reduced, while the remaining neuron parameters remain unchanged. We have demonstrated that this simplified model is able to reproduce the experimental results mentioned above (see Figure 8) as well as the model based on first order kinetics. This enabled us to obtain the mean contribution of a calcium spike to the somatic membrane potential, both semi-analytically and analytically. The analytical expressions improve on first order approximation by taking into account the covariance of synaptic conductances and membrane potential (see Figure 10).

The reduction of the first order kinetics to a threshold-triggered additive current is of a similar computational complexity as a common integrate-and-fire type model neuron. A difference is, though, that the model needs to store the empirically obtained waveform of the calcium current generated by the first-order kinetics. In terms of performance, this reduction is a considerable gain, which is evident by comparing the run times for simulating the three different variants of the model whilst applying a step current of 100pA to the soma for 100 s: (i) The model by Hay et al. (2011) simulated with the NEURON simulator (Carnevale and Hines, 2006) takes 6.15 s to run the simulation. (ii) The three compartment model with first-order kinetics implemented in NEST takes 2.15 s. The reduction of the first order kinetics to a threshold-triggered waveform reduces the simulation time to 1.04 s.

In the three compartment neuron model, the calcium spike is modeled at the distal compartment. As we are taking a reductionist approach in modeling the interacting dynamics of the calcium spike and the action potential generation with the aim to reproduce experimental results of action potentials and calcium spikes, we do not concern ourselves with microscopic properties of layer 5 pyramidal neurons, such as the distribution of calcium ion channels. These important characterizations remain open for future research.

The experimental results that we aim to reproduce in our model can be summarized as follows:

1. A large synaptic current at the distal compartment triggers a calcium spike that in turn triggers a burst of action potentials.
2. The distal current required for eliciting a calcium spike is considerably reduced if an action potential co-occurs.
3. A calcium spike does not result in a burst of action potentials if a hyper-polarizing current arrives at the proximal compartment.

While these by no means capture all experimental results of the calcium spike in the layer 5 pyramidal neuron [for instance, one very important result is that of critical frequency (Larkum et al., 1999b)], they are in our opinion sufficient to illustrate the role calcium spikes play in coincidence detection (both between an action potential at the soma and input at the distal compartment and multiple inputs at the distal compartments). While the two-compartment model by Larkum et al. (2004) reproduces some of these experimental results equally well, by construction, however, it cannot explain how a hyper-polarizing current at the proximal compartment prevents the calcium spike from triggering bursts at the soma. This is a crucial feature when investigating the role of calcium spikes e.g., in a model of a cortical column where synaptic inputs arrive at specific parts of the pyramidal neuron (Spruston, 2008).

In our analytical treatment to obtain the mean calcium somatic potential, a calcium threshold that deviates from the mean distal membrane potential results in an initial jump in the calcium somatic potential (see Figure 10B). This is intuitively explained by the conductances deviating from mean when the membrane potential is at the calcium threshold. Our analytical treatment hence accounts for the covariance of membrane potential and conductances in the mean but not when membrane potential and conductances are far from their respective mean values, which occurs at the calcium threshold. We have also not further extended our work to examine how the calcium somatic potential contributes to action potential generation. Such an attempt will have to take into account the adaptive threshold for action potentials.

Equipped with the presented reduced neuron model, in future work we may proceed to address earlier experimental findings in which a calcium spike generates burst of action potentials when triggered by coincident somatic and distal inputs (Larkum et al., 1999a) and the hypothesis that calcium spikes *in-vivo* may be involved in the detection of coincident inputs across compartments (Spruston, 2008). By introducing synchronous inputs at one or more of the compartments and systematically varying their magnitudes, while the neuron is embedded in background fluctuations, we can investigate the conditions under which calcium spikes are triggered and how they influence spiking activities.

Further work involving a network of these neurons can then be pursued to better understand its emergent properties. For example, different neurons are known to target different parts of the layer 5 pyramidal neuron. From Figure 1D, we know that a hyperpolarizing input at the proximal compartment will prevent a calcium spike from triggering action potential bursts. Hence, future work can investigate in network simulations how interneurons can be recruited by layer 5 pyramidal neurons to suppress other pyramidal neurons from bursting.

In Larkum et al. (2009), a 2 layered neuron model is proposed, where NMDA spikes from two or more compartments need to co-occur to trigger a calcium spike. Hence, calcium spikes may also be triggered by slow, local NMDA spikes. If the waveforms of NMDA-triggered calcium spikes are uniform, a similar analysis as presented here applies. To facilitate work in this direction, a neuron model with more compartments arranged in a 2 layered structure will have to be developed. The amount of synchronous inputs required to trigger an NMDA spike is also likely to be less than that required of calcium spikes. This would effectively make the neuron model more sensitive to synchronous inputs. The three compartment neuron model therefore helps to lay the groundwork necessary for future work involving other forms of dendritic spikes.

Our approach of introducing dendritic spikes in iso-potential compartment neuron models uses a relatively small number of variables (and thus can be simulated with moderate effort) and is not limited to single-cell studies but can be scaled up to understand the behavior of large-scale network models of neurons with dendritic spikes. This could lead to new insights, as typically studies of the interaction of structure and activity in large-scale neuronal networks have been limited to point neuron models (e.g., Kunkel et al., 2011; Potjans and Diesmann, 2014) or very complex neuron models (e.g., Hay et al., 2011; Bahl et al., 2012), whereas our model would allow the researcher to profit from both the speed and analytical tractability of the former approach and dynamic richness of the latter.

### Conflict of interest statement

The authors declare that the research was conducted in the absence of any commercial or financial relationships that could be construed as a potential conflict of interest.
