# Supplementary material for: Modeling the calcium spike as a threshold triggered fixed waveform for synchronous inputs in the fluctuation regime
Source: Front Comput Neurosci. 2015 Jul 28;9:91. doi: 10.3389/fncom.2015.00091 (PMC4516889; doi:10.3389/fncom.2015.00091)
Supplement: Supplementary file 1 [file Table1.PDF]

Neuron parameters		
Θ_{base}	base spike threshold	$-55.0\,\mathrm{mV}$
t_{ref}	refractory period	$2.0\,\mathrm{ms}$
V_{peak}	somatic membrane potential upon spike	$30.0\,\mathrm{mV}$
Θ_{+}	adaptive threshold jump	$6.0\,\mathrm{mV}$
τ_{th}	adaptive threshold time constant	$7.0\,\mathrm{ms}$
g_{sp}	leak across soma-proximal	$30.0\,\mathrm{nS}$
g_{pd}	leak across proximal-distal	$10.0\,\mathrm{nS}$
Calcium spike parameters		
U_{ca}	calcium reversal potential	$30.0\,\mathrm{mV}$
g_{ca}	calcium conductance	$70.0\,\mathrm{nS}$
τ_{m}	activating function time constant	$5.0\,\mathrm{ms}$
τ_{h}	deactivating function time constant	$50.0\,\mathrm{ms}$
m_{half}	activating function half voltage	$-21.0\,\mathrm{mV}$
h_{half}	deactivating function half voltage	$-24.0\,\mathrm{mV}$
m_{slope}	slope of activating function	0.5
h_{slope}	slope of deactivating function	-0.5
soma parameters		
$g_{\mathrm{l}}^{\mathrm{s}}$	leak during refractory period	$150.0\,\mathrm{nS}$
t_{ref}	duration of refractory period	$2.0\,\mathrm{ms}$
$g_{\mathrm{l}}^{\mathrm{s}}$	leak	$10.0\,\mathrm{nS}$
C^{s}	capacitance	$150.0\,\mathrm{pF}$
U_{l}^{s}	resting potential	$-70.0\,\mathrm{mV}$
$U_{\mathrm{e}}^{\mathrm{s}}$	excitatory reversal potential	$0.0\,\mathrm{mV}$
$U_{\mathrm{i}}^{\mathrm{s}}$	inhibitory reversal potential	$-85.0\,\mathrm{mV}$
$\tau_{\mathrm{e}}^{\mathrm{s}}$	excitatory synaptic time constant	$1.0\,\mathrm{ms}$
$\tau_{\mathrm{i}}^{\mathrm{s}}$	inhibitory synaptic time constant	$2.0\,\mathrm{ms}$
proximal parameters		
$g_{\mathrm{l}}^{\mathrm{p}}$	leak	$10.0\,\mathrm{nS}$
C^{p}	capacitance	$80.0\,\mathrm{pF}$
$U_{\mathrm{l}}^{\mathrm{p}}$	resting potential	$-65.0\,\mathrm{mV}$
$U_{\mathrm{e}}^{\mathrm{p}}$	excitatory reversal potential	$0.0\,\mathrm{mV}$
$U_{\mathrm{i}}^{\mathrm{p}}$	inhibitory reversal potential	$-85.0\,\mathrm{mV}$
$\tau_{\mathrm{AP}}^{\mathrm{p}}$	time constant of bpAP alpha current during spike	$1.0\,\mathrm{ms}$
$J_{\mathrm{AP}}^{\mathrm{p}}$	amplitude of bpAP alpha current	$400.0\,\mathrm{pA}$
$\tau_{\mathrm{e}}^{\mathrm{p}}$	excitatory synaptic time constant	$1.0\,\mathrm{ms}$
$\tau_{\mathrm{i}}^{\mathrm{p}}$	inhibitory synaptic time constant	$2.0\,\mathrm{ms}$
distal parameters		
$g_{\mathrm{l}}^{\mathrm{d}}$	leak	$20.0\,\mathrm{nS}$
C^{d}	capacitance	$60.0\,\mathrm{pF}$
$U_{\mathrm{l}}^{\mathrm{d}}$	resting potential	$-60.0\,\mathrm{mV}$
$U_{\mathrm{e}}^{\mathrm{d}}$	excitatory reversal potential	$0.0\,\mathrm{mV}$
$U_{\mathrm{i}}^{\mathrm{d}}$	inhibitory reversal potential	$-85.0\,\mathrm{mV}$
$\tau_{\mathrm{AP}}^{\mathrm{d}}$	time constant of bpAP alpha current during spike	$1.0\,\mathrm{ms}$
$J_{\mathrm{AP}}^{\mathrm{d}}$	amplitude of bpAP alpha current	$310.0\,\mathrm{pA}$
$\tau_{\mathrm{e}}^{\mathrm{d}}$	excitatory synaptic time constant	$1.0\,\mathrm{ms}$
$\tau_{\mathrm{i}}^{\mathrm{d}}$	inhibitory synaptic time constant	$2.0\,\mathrm{ms}$

Table S1: Parameters of the neuron model with first order kinetics calcium spike. The action potential is modeled by a jump in membrane potential and a large leak during refractory period while the threshold is adaptive. Alpha currents (2) are added at the proximal and distal compartment 1 and 2 ms after spike to emulate a back-propagating action potential.
